# Comparison of Error Incidence Between Single-Tablet Versus Multiple-Tablet INSTI-Based Regimens in the Inpatient Setting

**DOI:** 10.1177/10600280251324337

**Published:** 2025-03-21

**Authors:** Denise Kelley, Kayla Blackmon, Brian L. Nguyen, Dusten T. Rose

**Affiliations:** 1 University of Texas, College of Pharmacy, Austin, TX, USA; 2 Department of Pharmacy, Ascension Seton, Dell Seton Medical Center at the University of Texas, Austin, TX, USA; 3 Department of Pharmacy, AscensionConnect GoodHealth Solutions Center, Austin, TX, USA

**Keywords:** HIV/AIDS, integrase strand transfer inhibitors, antiretrovirals, medication errors, hospital pharmacy

## Abstract

**Background:**

Errors related to antiretroviral therapy (ART) occur in up to 86% of hospitalized patients living with human immunodeficiency virus (HIV) and may contribute to treatment failure, drug resistance, adverse effects, and toxicity. ART can be administered as a single-tablet regimen (STR) or multiple-tablet regimen (MTR), with limited data on whether the number of tablets affects inpatient error incidence.

**Objective:**

The purpose of this study was to determine the error rate of substituting dolutegravir-based STRs to an MTR while admitted.

**Methods:**

This multicenter, retrospective, observational study in adult inpatients receiving ART for HIV evaluated continuation of bictegravir-based STR versus dolutegravir-based STR given as an MTR. The primary outcome was the composite error incidence when ART was dispensed as an STR versus MTR. Secondary endpoints included number of errors per patient encounter, between-group error types, time to error correction and pharmacist involvement, and conversion back to STR at discharge.

**Results:**

Of 514 patient encounters (257 bictegravir-based STR; 257 dolutegravir-based MTR), there was a significantly lower composite incidence of errors in the STR group versus the MTR group (23% vs 31.5%; *P* = 0.029). A significantly higher incidence of dose-related errors in the MTR group occurred related to renal or hepatic dose adjustments, which was the only significantly different between-group error type identified. Approximately one error per encounter was identified in both groups, with median time to error correction slightly over 1 day. Multiple-tablet regimens were converted back to an STR at discharge in 89.9% of admissions.

**Conclusion and relevance:**

Providing INSTI-based ART as an STR while admitted may reduce ART-related medication errors and has potential to improve patient care; however, use of an STR may not address errors related to inappropriate dosing in organ dysfunction. Increased vigilance for medication errors is warranted when substituting with MTRs in inpatient settings.

## Introduction

With more than 1 million individuals living with human immunodeficiency virus (HIV) in the United States, and an estimated 31 800 new HIV diagnoses in 2022, the number of persons with HIV (PWH) on antiretroviral therapies (ARTs) continues to increase.^1^ In the study site region (Austin, Texas), the HIV prevalence rate in 2022 was reported as slightly higher than the United States overall (398 vs 388 per 100 000 patients).^2^ Given the heightened risk for ART-related errors during hospitalization, and rates widely ranging between 5% and 86%, inpatient antiretroviral stewardship is a key component for providing quality care to this population and ensuring patient safety.^3-5^ ART–related errors have the potential to contribute to treatment failure, increased drug resistance, adverse effects, and toxicity. The most commonly reported errors include incomplete regimen or dose omission, incorrect dose or schedule, lack of dose adjustment for organ dysfunction, and clinically significant drug-drug interactions (DDIs).^6-8^ Interruptions in treatment are associated with rebound viremia, worsening immune function, and increased morbidity and mortality.^9^ The resolution of ART-related errors remains a primary focus in promoting antiretroviral stewardship, and is an area where clinical pharmacists can have a substantial impact.

Many studies have previously examined the impact of antiretroviral stewardship, primarily describing the success of local interventions at single centers utilizing varying resources (eg, infectious diseases pharmacy specialists, pharmacy residents, or clinical decision support tools).^10,11^ Identifying what ART characteristics may lead to more potential errors is less clear, although some have identified the use of protease inhibitors (PIs) as contributing to increased error rates mostly due to clinically significant DDIs.^4,12^ One study attributed prescribing errors to lack of available coformulated products of nucleoside and non-nucleoside reverse transcriptase inhibitors (NRTIs and NNRTIs) increasing the likelihood of drug omission, while another saw increased errors with the use of non-formulary ART.^12,13^ Error rates for integrase strand transfer inhibitors (INSTIs) are less well documented, although appear to be lower than other ART types.^12,14^

Most practitioners reference lack of experience and comfortability with ART as leading contributors to high error rates observed. This is further complicated by rapidly evolving treatment recommendations and patient-specific information that may be unavailable to inpatient providers during admission. Many medications are coformulated into single-tablet regimens (STRs) to offer improvements in patient quality of life, adherence, and hospitalization rates, which when continued in the inpatient setting can pose a challenge to the healthcare system in maintaining each STR on formulary.^15^ Many hospitals attempt to reduce formulary expenses by dispensing the STR as individual components when able, only stocking a specific STR when there is no alternative. While coformulated tablets may present a cost barrier to hospital formularies, the high error rate presents an additional cost burden. One academic medical center reported a potential cost savings of $260 000 annually, achieved by the antiretroviral stewardship team’s contributions to error resolution (mean $813/intervention).^16^ This study also identified multiple-tablet regimens (MTRs) as a risk factor for inpatient ART-related errors.^16^ Although substituting MTR for STR may be a cost-containment practice, little is known about the clinical impact or potential for harm. The purpose of this study is to compare composite error incidence in hospitalized adults who received bictegravir-based STR versus those substituted from a dolutegravir-based STR to an MTR while admitted.

## Materials and Methods

A multicenter, retrospective observational study was conducted for adult PWH admitted to an Ascension Seton hospital from January 1, 2017, through September 1, 2022, and receiving an ART regimen consisting of either bictegravir-based STR or dolutegravir-based STR.^17^ This study was approved by the Ascension Seton Institutional Review Board. Encounters from 9 hospital sites were evaluated, including community-based practice sites and 1 academic teaching hospital. All hospital sites have access to a comprehensive local guideline with a verification checklist for pharmacists including renal and hepatic dosing, guidance on assessing patient compliance, DDIs, and formulary management. In addition, all patient encounters with active ART orders are flagged for evaluation and reviewed by a floor-based clinical pharmacist daily.

**Figure 1.**
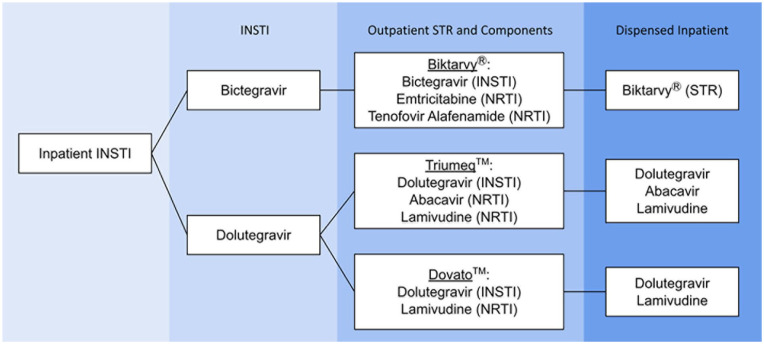
Inpatient Processing of INSTI Antiretrovirals. Abbreviations: INSTI, integrase strand transfer inhibitor; NRTI, nucleotide reverse transcriptase inhibitor.

Encounters of hospitalized patients were included if patients were ≥18 years, had a diagnosis of HIV prior to admission, were prescribed a bictegravir-based or dolutegravir-based STR prior to admission, and had inpatient orders for either a bictegravir-based STR (Biktarvy) or a dolutegravir-based MTR as a substitute for dolutegravir and lamivudine (Dovato) or dolutegravir, lamivudine, and abacavir (Triumeq) (Figure 1). This study intentionally excluded the dolutegravir-rilpivirine STR (Juluca) as it has notable differences that could unfairly bias the outcomes of the study (eg, contains NNRTI rather than NRTI and additional food and DDI concerns). The study period for patients in the MTR arm was from January 1, 2017, through September 1, 2022; however, all patient encounters included in the STR arm were admitted after May 1, 2020, following the formulary addition of bictegravir-based STR. Patients were excluded if pregnant, incarcerated, not taking ART prior to admission, or admitted for <24 hours and discharged prior to receiving any ART doses. Patients may have had multiple encounters throughout the study period, as each admission was individually evaluated.

The primary outcome of this study was a comparison of composite error incidence in patient encounters continued on home bictegravir-based STRs versus those substituted from a dolutegravir-based STR to an MTR while admitted. Identified errors were classified as incorrect regimen, clinically significant DDIs, incorrect dose, or incorrect frequency. If at least one error type occurred in a patient encounter, the encounter was marked “yes” for error incidence. Incorrect regimen consisted of therapeutic duplications, erroneous antiretroviral agents ordered, omitted doses (doses not ordered within 24 hours of admission and/or any doses not administered despite an active order due to medication not being available or patient refusal), and incomplete regimens. Incorrect doses were defined as dosing not in accordance with the Department of Health and Human Services (DHHS) HIV guidelines, including dosing too high or too low in renal (emtricitabine, lamivudine) or hepatic (abacavir) impairment.^9^ The study site’s local guideline recommended against using Biktarvy if estimated creatinine clearance (eCrCl) fell below 30 mL/min. Recommendations for lamivudine renal dose adjustments based on eCrCl vary depending on source; while lamivudine’s package insert advises reducing doses if eCrCl <50 mL/min, DHHS guidelines use a threshold of <30 mL/min.^9,18^ An eCrCl of <30 mL/min was used to identify dosing errors related to renal function in this study, as this also aligned with the hospital’s local guideline. If lamivudine was dose reduced for eCrCl <50 mL/min, but >30 mL/min, it was considered an inappropriately low dose. Secondary outcomes included number of errors per encounter among those with at least 1 error, between-group error types, time to error correction, correct conversion from MTR to STR at discharge, and pharmacist involvement in error correction. In addition, a cost analysis was performed to evaluate the anticipated cost of STR acquisition for the 2 dolutegravir-based regimens included in this study using acquisition wholesale price (AWP). A dispense report for the year 2022 was used to approximate annual usage of both regimens.

### Statistical Analysis

A sample size of 514 was determined to achieve 80% power in detecting an 11% difference in error incidence, assuming a baseline error incidence of 22% for inpatient ART. The primary outcome was evaluated using a χ^2^ test. Among secondary outcomes, a χ^2^ test was used to evaluate between-group error types for each of the 4 error categories and a Wilcoxon rank sum was used for nonparametric data (time to error correction). All other secondary outcomes were evaluated with descriptive statistics.

## Results

**Figure 2.**
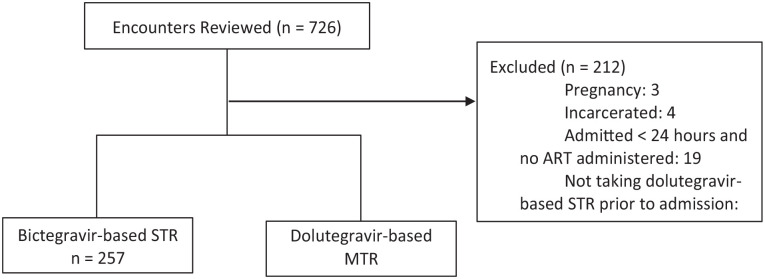
Enrollment.

Of 726 patient encounters screened, 514 were included in this study (n = 257, bictegravir STR; n = 257, dolutegravir MTR). The most common reason for exclusion was not taking a dolutegravir-based STR prior to admission (186/212, 87.7%) (Figure 2). Patient encounters had a mean age of 48 years (±12.6) and were mostly male (79%). Median weight, baseline renal function assessed by serum creatinine, and baseline history of cirrhosis were similar between groups (Table 1). Outpatient ART regimens in the dolutegravir-based group mostly consisted of the 3-drug regimen, Triumeq (89.9%), followed by Dovato (10.1%). The composite error incidence occurred in significantly less patient encounters in the STR group compared with the MTR group (59/257, 23% vs 81/257, 31.5%, *P* = 0.029). Of the total errors identified (STR 68 vs MTR 101), the most common error type for both groups was incorrect regimen (STR 41/68, 60.3% vs MTR 46/101, 45.5%), specifically related to dose omissions comprising 82.9% and 73.9% of these errors, respectively. Dose omissions were primarily driven by missing initial doses upon admission. The only error type that significantly differed was an increased incidence of incorrect dose in the MTR arm (STR 0/68, 0% vs 28/101, 27.7%). A total of 21 patient encounters were found to only have an error classified as incorrect dose, and when removed from the analysis, no difference in composite error incidence was seen between the STR and MTR groups (59/257, 23.0% vs 60/257, 23.3%, *P* = 0.917). Correct conversion from MTR back to STR at discharge occurred in 89.9% of patient encounters, and this outcome was not applicable to assess in the bictegravir-based arm. All other outcomes were similar between groups (Tables 2 and 3). When considering AWP of the individual components of the dolutegravir-based MTRs evaluated compared with the corresponding STR, the anticipated cost of acquiring the STRs was found to be $6872.46 more per year based on usage observed in 2022 (Table 4).

**Table 1.** Baseline Characteristics.

Characteristics	STR (n = 257)	MTR (n = 257)
Age, years, mean (±SD)	46.6 (±12.6)	50.2 (±12.6)
Male sex, no. (%)	196 (76)	211 (82)
Weight, kg, median (IQR)	76 (67-89)	84 (72-97)
Baseline serum creatinine, mg/d, median (IQR)	1 (0.8-1.2)	1.1 (0.9-1.4)
Peak serum creatinine, mg/dL, median (IQR)	1.1 (0.9-1.3)	1.2 (0.9-1.5)
Baseline eCrCl, mL/min, median (IQR)	81 (63-103)	74 (51-98)
Lowest eCrCl, mL/min, median (IQR)	76 (57-99)	72 (48-95)
Child-Pugh Score		
No history of cirrhosis, no. (%)	246 (95.7)	232 (90.3)
Class A, no. (%)	5 (1.9)	10 (3.9)
Class B, no. (%)	4 (1.6)	15 (5.8)
Class C, no. (%)	2 (0.8)	0 (0)
Outpatient STR Regimen		
Biktarvy (BIC-FTC-TAF), no. (%)	257 (100)	0 (0)
Triumeq (DTG-ABC-3TC), no. (%)	0 (0)	231 (89.9)
Dovato (DTG-3TC), no. (%)	0 (0)	26 (10.1)

Abbreviations: eCrCl, estimated creatinine clearance; STR, single-tablet regimen; BIC, bictegravir; FTC, emtricitabine; TAF, tenofovir alafenamide; DTG, dolutegravir; ABC, abacavir; 3TC, lamivudine.

**Table 2.** Primary and Secondary Outcomes.

Outcome	STR (n = 257)	MTR (n = 257)	*P* value
**Primary outcome**			
Composite error incidence, no. (%)	59 (23)	81 (31.5)	0.029
**Secondary outcomes**			
Composite error incidence excluding incorrect dose, no. (%)	59 (23)	60 (23)	0.917
Number of errors per encounter (among those with ≥ 1 error), mean (± SD)	1.15 (±0.45)	1.22 (±0.52)	–
Between group error type			
Incorrect regimen, no. (%)	41 (15.9)	36 (14)	0.918
Drug-drug interaction, no. (%)	27 (10.5)	26 (9.7)	0.869
Incorrect dose, no. (%)	0 (0)	37 (14.4)^a^	<0.001
Incorrect frequency, no. (%)	0 (0)	1 (0.4)	0.499
Time to error correction, hours, median (IQR)	28.3 (20-58)	26 (18-67)	0.748
Correct conversion from MTR to STR at discharge, no. (%)	–	231 (89.9)	–
Pharmacist involved in error correction, no. (%)	14 (20.6)	21 (21.2)	–

a Refers to total number of errors meeting study definition of incorrect dose.

**Table 3.** Breakdown of Error Types.

Error Type	STR (n = 68)	MTR (n = 101)
**Incorrect regimen, no. (%)**	41 (60.29)	46 (45.54)
Therapeutic duplication, no. (%)	2 (2.94)	5 (4.95)
Erroneous ART ordered, no. (%)	5 (7.35)	4 (3.96)
Omitted dose, not ordered, no. (%)	15 (22.06)	12 (11.88)
Omitted dose, not administered, no. (%)	19 (27.94)	22 (21.78)
Incomplete regimen, no. (%)	–	3 (2.97)
**Drug-drug interaction, no. (%)**	27 (39.71)	26 (25.74)
Contraindicated, no. (%)	4 (5.88)	3 (2.97)
Anti-seizure, no. (%)	3 (4.41)	3 (2.97)
Rifamycins, no. (%)	1 (1.47)	0 (0)
Administration-time dependent, no. (%)	23 (33.82)	23 (22.77)
Antacids, no. (%)	18 (26.47)	23 (22.77)
Calcium/iron-containing supplements, no. (%)	5 (7.35)	0 (0)
**Incorrect dose, no. (%)**	–	28 (27.72)
Not renally dose reduced, dose too high, no. (%)		10 (9.90)
Not renally dose adjusted, dose too low, no. (%)		4 (3.96)
Not hepatically dosed, dose too high, no. (%)		14 (13.86)
**Incorrect frequency, no. (%)**		1 (0.99)
Not once daily, no. (%)		0 (0)
Not twice daily, no. (%)^a^	–	1 (0.99)

a One patient on dolutegravir merited twice daily dosing due to a concomitant DDI.

**Table 4.** Cost Analysis Based on Acquisition Wholesale Prices (AWP) for STR Versus MTR.

	AWP per dose (MTR)	AWP per dose (STR)	Difference per dose	Annual cost (MTR)^a^	Annual cost (STR)^a^	Annual difference
**Triumeq** DTG 3TC ABC	**$119.64** $85.23 $14.32 $20.09	**$141.50**	**+$21.86**	**$30 867.12** $21 989.34 $3694.56 $5183.22	**$36 507.00**	**+$5639.88**
**Dovato** DTG 3TC	**$99.55** $85.23 $14.32	**$112.39**	**+$12.84**	**$9556.80** $8182.08 $1374.72	**$10 789.38**	**+$1232.58**

Abbreviations: MTR, multiple-tablet regimen; STR, single-tablet regimen; DTG, dolutegravir; 3TC, lamivudine; ABC, abacavir.

a Based on the health system’s inpatient usage from 2022.

## Discussion

This is the first study, to the authors’ knowledge, to evaluate inpatient medication errors as it relates to the formulary practice of substituting INSTI-based STR to individual components. It also adds to the limited literature quantifying the proportion of inpatient errors associated with INSTI-based regimens. Our data demonstrate an advantage of STRs over MTRs in reducing the composite error incidence among hospitalized PWH. This was driven by the difference in incidence of incorrect dose in the MTR arm, and when this error type was removed from the composite outcome, no difference was found between the 2 arms.

In evaluating incorrect dosing error types, 28 were related to missed renal or hepatic dosing adjustments, which disproportionately affected the MTR arm. All patients in the STR and MTR arm received an agent where renal dosing may be considered (emtricitabine or lamivudine, respectively) while nearly 90% in the MTR arm received abacavir which has dosing recommendations in hepatic impairment. Renal and hepatic function were similar at baseline between treatment arms, so it was not anticipated to see many dosing errors related to organ dysfunction; however, acute organ dysfunction in the inpatient setting can be common, particularly in hospitalized PWH, with incidence of acute kidney injury (AKI) previously reported in 18% of patients.^19^

When emtricitabine is given as a component of Biktarvy, no dose adjustment is recommended if eCrCl >30 mL/min.^9^ While the recommendations are the same with lamivudine when given as Triumeq or Dovato, this conflicts with the labeling for individual lamivudine.^18^ Data for lamivudine in renal impairment were originally derived from pharmacokinetic studies, which demonstrated increased levels of lamivudine with decreasing renal function, as ~70% of orally administered drug is renally excreted.^20^ However, the favorable safety profile, even despite increased levels, and potential consequences of using an MTR in the outpatient setting (eg, increased pill burden, reduced adherence), create controversy around the need for lamivudine dose adjustments in renal impairment.^20,21^ Given the interquartile range of lowest eCrCl in the MTR group at baseline was 48 mL/min, renal dosing errors were likely most commonly identified in the setting of AKI, which may not indicate a chronic adjustment in dosing. Of the 28 dosing-related errors in the MTR group, 14 (50%) were inappropriately renally dosed with 4 of these encounters occurring in the setting of eCrCl <50 mL/min, rather than eCrCl <30 mL/min, which is an error based on the study site’s internal guidance but not necessarily an error depending on the resource.

There were 14 abacavir dosing errors in the setting of hepatic dysfunction, which may be limited by the evaluation of Child-Pugh scores, as our baseline assessment would suggest even home doses were not appropriately adjusted. Similar to lamivudine, available evidence supporting the need to dose adjust is limited, with only pharmacokinetic studies available demonstrating 89% increase in AUC and 58% increase in half-life in patients with mild hepatic dysfunction (Child-Pugh score 5-6).^22^ Achieving a hepatic dose of abacavir may also be impractical based on dosage forms available and inadvertently lead to negative downstream effects. While concomitant cirrhosis in PWH has been found to be as high as 11.4%, performing a Child-Pugh score via retrospective chart review may falsely yield results of Child-Pugh B or C in a non-cirrhotic patient, implying a dose reduction is needed when it may not actually be necessary, which could have contributed to the findings of this study and should be taken lightly.^23^

The incidence of dosing errors, specifically related to dose adjustments in organ dysfunction, drove the composite outcome in this study, and does not fairly shed light on the comparison of STR versus transition to MTR in the inpatient setting as it more so compares dosing nuances between the NRTIs in each regimen. In addition, if dose adjustment was truly indicated given the respective clinical scenario, a reduction from the fixed-dose combination in STRs would have been warranted regardless of whether the STR is on the hospital formulary. One patient in the MTR arm merited appropriate adjustment to twice daily dolutegravir while admitted due to a concomitant DDI and would have also precluded the use of an STR.

One notable finding that does directly compare the STR versus transition to MTR is in the correct conversion from MTR back to the patient’s original STR at discharge. This revealed an unexpectedly higher proportion of encounters that were not appropriately discharged on their original STR prior to admission. While 2 patients were appropriately discharged on an MTR due to the need for renal dose adjustments, a large proportion of these encounters (16/26, 61.5%) were discharged with a documented ART regimen consisting of multiple tablets. Of those, 5 encounters had prescriptions sent to be filled for the MTR. Approximately one fourth (6/26, 23.1%) of these encounters had a discharge medication list without any ART regimen documented, despite clinical documentation supporting the plan to continue therapy. The process for discharge medication reconciliations at the study site allows providers to select both home and inpatient medications for continuation, which may have led to inadvertent continuation of MTR regimens. While most patients did not appear to receive a new prescription for the documented MTR, these medication lists persist in the electronic health record (EHR) and may lead to errors during future admissions. Timely review of medications at discharge is warranted, particularly in health systems that substitute STRs with MTRs while admitted.

Even when removing incorrect dose from the total incidence of errors, a higher numerical error rate occurred in the MTR group which, while likely multifactorial, still warrants attention. Interestingly, the STR arm had a higher numerical incidence of incorrect regimen, primarily due to omitted dose, not ordered or not administered. It is unclear how to explain this finding (chance, enteral access issues in the STR arm, operational workflow with Biktarvy following initial formulary approval) but deserves further exploration. Overall composite error incidence per patient encounter was ~27%, which is comparable with previously reported ART error rates (approximately 16.7% to 54.7% of patient admissions).^6,7,16,24^ In addition, our breakdown of error types is similar to prior studies, with incorrect regimen most commonly reported and incorrect schedule least commonly reported.^6,7,24,25^ Among patient encounters with at least one error identified, the average number of errors per encounter were similar to outcomes in existing literature, which demonstrated a range of 1.5 to 2.4.^6,7,24^ The lower error incidence per patient encounter in this study is likely attributable to differences in ART component type. Studies evaluating error incidence specifically among INSTI-based regimens are lacking with one small study reporting a 13% error rate in 20 patients receiving raltegravir. Similar to our study, their most common error types were omitted order (50%) and incorrect dose (45%); however, fewer DDIs were identified.^12^ Despite NRTIs and INSTIs leading to significantly fewer errors compared with higher-risk components, like PIs, this study demonstrates the need for caution as INSTI-based regimens remain a significant risk for errors in inpatient settings.^12^ Median time to error correction just exceeded 24 hours, and while this length of time is comparable across studies, it suggests remaining opportunities for improvement. Pharmacist involvement in error resolution was identified in only 21% of errors; however, this rate may be falsely low as our process for identifying pharmacist involvement excluded an additional platform where many interventions are documented and may have missed interventions made verbally or without formal documentation.

This study had notable limitations, such as its retrospective design and inability to assess potential downstream effects associated with ART-related errors. A significant portion of the literature evaluating inpatient ART-related errors is retrospective given the need to evaluate for errors, which are unintended outcomes of the inpatient medication use process that are less likely to occur under controlled conditions. Capturing events retrospectively posed a challenge in some cases, specifically omitted doses, not ordered and omitted doses, not administered. There could be plausible explanations for why the dose was omitted (eg, omitted on day of admission if the patient had previously taken at home) or true findings (eg, patient refusal of MTR due to confusion knowing the usual regimen is an STR). An estimated baseline error incidence of 22%, though lower than some reported incidences in literature, was used given the smaller pool size of ART evaluated and the decreased risk of DDIs anticipated with INSTI-based regimens. With limited historical data evaluating INSTI-based ART specifically, an 11% difference between STR and MTR arms was conservatively anticipated based on a prior study showing an approximately 7% to 17% error reduction attributed to coformulated tablets when compared with PI and NRTI rates.^13^ This may have added limitations to the study by allowing for a smaller sample size to meet power; however, the primary outcome demonstrated an error incidence in line with this estimate, and our study was adequately powered based on the sample size calculated. Strict clinical definitions for organ dysfunction were used, and this study may represent a conservative/potential overestimate of clinical consequences due to ART-related errors in this population, although definitions adhered to DHHS dosing recommendations.^9^ In addition, this study did not evaluate all potential INSTI-based STRs, as dolutegravir-rilpivirine (Juluca) and elvitegravir-containing STR (Genvoya, Stribild) were intentionally excluded in an attempt to minimize unfairly observing more DDI errors.

The calculated cost difference to dispense the STRs Triumeq and Dovato was an additional $7000/year. While this increase in acquisition cost is likely less than the potential savings of reducing errors, the value in making this formulary change may not necessarily be supported based on the results of this study.^16^ As generic equivalents are brought to market and comparability of cost between STRs and MTRs evolves, additional consideration may be warranted.

## Conclusion and Relevance

This study provides valuable insight to the incidence of inpatient errors associated with INSTI-based regimens. With increasing availability of fixed-dose combination tablets (and majority of INSTI-based regimens available as STRs), there is increasing difficulty for health systems to maintain all available ARTs, often substituting STR to MTR while admitted when able. The current formulary practice of dispensing dolutegravir-based STRs as MTR while admitted may be an acceptable practice, although increased vigilance is still warranted for errors related to dosing for organ dysfunction and at hospital discharge.
